# Digital Health Technologies for Diabetic Foot Ulcers: A Systematic Review of Clinical Evidence, Access Inequities, and Public Health Integration

**DOI:** 10.3390/ijerph22091430

**Published:** 2025-09-13

**Authors:** Tatiana Cristina Dias de Oliveira, Alana Ferreira de Oliveira, Laila de Castro Araújo, Maria Pantoja Moreira de Sena, Valéria de Castro Fagundes, Phelipe Augusto Rabelo Paixão, Stefani Gisele Bastos Dornas, Clarisse Andrade Sales, Ana Paula Simões Castro, Patricia Alves de Mendonça Cavalcante, Luann Wendel Pereira de Sena

**Affiliations:** 1 Graduate Program in Family Health, Federal University of the South and Southeast of Pará, Maraba 68507-590, Brazil; 2 Postgraduate Program in Tropical Diseases, Federal University of Pará, Belem 66075-110, Brazil; 3 Faculty of Medical Sciences of Pará, Para 38405-320, Brazil; 4 Postgraduate Program in Biodiversity and Biotechnology, Federal University of the South and Southeast of Pará, Maraba 68507-590, Brazil

**Keywords:** diabetic foot, health technology, mobile health applications, digital health

## Abstract

Diabetic foot ulcers are among the most severe complications of diabetes mellitus, disproportionately affecting populations in low- and middle-income countries. Digital health technologies have emerged as promising tools for prevention, diagnosis, and management; however, their effectiveness, usability, and applicability within public health systems remain insufficiently defined. This systematic review aimed to critically synthesize the clinical effectiveness, perceived usability, and methodological quality of digital interventions for the care of individuals with diabetes-related foot ulcers. A comprehensive search was performed in PubMed, Scopus, Web of Science, Embase, and Google Scholar for studies published between 2012 and 2024. Eighteen studies met the inclusion criteria, encompassing mobile health applications, wearable sensor devices, artificial intelligence-based tools, and telehealth platforms. Methodological quality was assessed using the Mixed Methods Appraisal Tool. Artificial intelligence-driven approaches demonstrated high diagnostic accuracy, with sensitivity and specificity above 90% for ulcer detection and classification. Mobile applications showed positive effects on self-efficacy, glycemic control, and adherence to preventive foot care, while usability scores were consistently high. Wearable sensor devices demonstrated potential for reducing ulcer recurrence, though supporting evidence remains limited. Across studies, recurrent methodological limitations included small sample sizes, absence of control groups, lack of economic evaluations, and barriers related to digital literacy and interoperability between systems. Most investigations were conducted in high-income countries, with limited consideration of public health contexts such as the Brazilian Unified Health System. In conclusion, digital health technologies show promise in improving the care of individuals with diabetes-related foot complications but face significant challenges regarding scalability, equity of access, and integration into public healthcare systems. Future research should prioritize context-adapted designs, robust clinical trials, and economic evaluations to inform health policies and support the rational adoption of these tools within universal health coverage frameworks. PROSPERO registration number: CRD420251023152.

## 1. Introduction

Diabetic foot ulcers (DFU) are among the most severe and recurrent complications of diabetes mellitus, affecting 15–25% of patients during their lifetime [1]. They account for up to 85% of non-traumatic lower-limb amputations and are closely linked to functional decline, increased mortality, and substantial economic burden, particularly in low- and middle-income countries (LMICs) [2,3].

Despite advances in management—including surgical debridement, advanced wound dressings, and intensive glycemic control—recurrence rates remain high, with nearly 40% of patients experiencing a new ulcer within one year of initial healing [4]. In health systems with limited infrastructure and workforce shortages, such as those in parts of Latin America, these challenges are compounded by barriers to early diagnosis, continuity of care, and patient education.

In response, digital health technologies have gained prominence in clinical practice. These include mobile applications for self-care, wearable sensors for early detection of plantar changes, artificial intelligence (AI)-driven systems for automated triage, and telehealth platforms for remote monitoring [5,6,7]. When effectively implemented, such tools can expand access to healthcare, reduce hospitalizations, and strengthen disease surveillance in both urban and rural populations.

However, the literature demonstrates marked heterogeneity in the technologies evaluated, the outcomes measured, and the methodological quality of studies [8,9]. Moreover, most innovations are developed in high-income countries (HICs) and often fail to address structural barriers typical of public health systems, including low digital literacy, poor internet connectivity, fragmented electronic health records, and limited regulatory frameworks for digital innovation.

Thus, beyond clinical efficacy, it is essential to examine how digital interventions interact with public health governance, resource allocation, and equity of access—particularly within universal health systems such as the Brazilian Unified Health System (SUS). As digital health strategies become central to chronic disease management, their implementation must be critically appraised in terms of system capacity, workforce readiness, and the ethical implications of technological exclusion.

Against this backdrop, the present systematic review critically synthesizes current evidence on digital health technologies for the prevention, monitoring, and treatment of DFU. The review emphasizes clinical effectiveness, usability, and methodological rigor, while also addressing broader implications for access, equity, and health system integration in resource-constrained contexts.

## 2. Methods

This systematic review was conducted in accordance with the Preferred Reporting Items for Systematic Reviews and Meta-Analyses (PRISMA 2020) guidelines. The protocol was prospectively registered in the PROSPERO database (CRD420251023152). All methodological steps were performed independently by two trained reviewers to ensure transparency, reproducibility, and methodological rigor. Discrepancies were resolved by consensus with a third reviewer [10].

### 2.1. Data Sources and Search Strategy

A comprehensive literature search was conducted in PubMed/MEDLINE, Scopus, and Web of Science. The search covered studies published between 1 January 2012, and 15 April 2025, reflecting a 13-year period of rapid expansion in digital health technologies applied to diabetic foot care.

The search strategy combined controlled vocabulary (Medical Subject Headings [MeSH] and Health Sciences Descriptors [DeCS]) with free-text terms, organized into three domains: (i) clinical condition (“diabetes mellitus,” “diabetic foot,” “foot ulcer”); (ii) digital technologies (“mobile applications,” “electronic health,” “mobile health,” “artificial intelligence,” “wearable sensors”); and (iii) intervention functionalities (“self-care,” “health education,” “remote monitoring,” “psychometric validation”). Boolean operators (“AND,” “OR”) were used to combine terms, and the syntax was adapted to each database. The complete search strategy is provided in Supplementary Material File S1.

Gray literature was also screened through Google Scholar, restricted to the first 60 results ranked by relevance. Reference lists of included articles were manually examined to identify additional eligible studies, and the ClinicalTrials.gov registry was consulted. Non-peer-reviewed documents (e.g., preprints, patents, dissertations, and technical reports) were excluded.

### 2.2. Eligibility Criteria

This review included empirical studies with human participants diagnosed with type 1 or type 2 diabetes mellitus, evaluating digital health technologies for the prevention, detection, monitoring, education, or remote support of plantar foot ulcers. Eligible interventions comprised mobile health applications, wearable sensors, artificial intelligence (AI)-based systems, digital platforms, and clinical decision-support tools.

Studies were required to report at least one clinical outcome (e.g., ulcer incidence or recurrence, wound-healing time, glycemic control) or one psychometric outcome (e.g., usability, self-efficacy, adherence to foot care, technology acceptance). Only articles published between January 2012 and April 2025 were considered.

Exclusion criteria included publications not in English, Portuguese, or Spanish; literature reviews, editorials, letters, or conference abstracts; studies using animal models or computer simulations without clinical validation; and studies lacking measurable clinical or psychometric outcomes.

### 2.3. Study Selection

The study selection followed three sequential steps: (i) removal of duplicates, (ii) screening of titles and abstracts, and (iii) full-text assessment. The Rayyan QCRI platform was used to enable blinded screening and decision tracking [11]. Two independent reviewers (T.C.D.d.O. and A.F.d.O.) performed the selection, achieving a Cohen’s kappa coefficient of 0.84, which indicates substantial agreement. Discrepancies were resolved by a third reviewer (L.W.P.d.S.). When full texts were unavailable, corresponding authors were contacted via institutional email or through the ResearchGate platform.

### 2.4. Data Extraction

Data extraction was conducted using a structured Microsoft Excel^®^ spreadsheet, pilot-tested on five randomly selected studies. Two reviewers (T.C.D.d.O. and A.F.d.O.) independently extracted the data, and discrepancies were resolved through discussion with a third reviewer (L.W.P.d.S.).

Extracted variables included study identification (first author, year), country of origin, study design, sample characteristics, type of digital technology, clinical and operational outcomes, psychometric instruments, and main findings.

### 2.5. Methodological Quality Assessment

The methodological quality of included studies was assessed using tools appropriate to each study design. Randomized controlled trials were evaluated with the Cochrane Risk of Bias 2.0 (RoB 2.0), which covers randomization, deviations from intended interventions, missing data, outcome measurement, and reporting bias. Non-randomized studies were assessed with the Risk Of Bias In Non-randomized Studies of Interventions (ROBINS-I), which examines seven domains including confounding, participant selection, and selective reporting.

Observational studies were appraised using the Newcastle–Ottawa Scale (NOS), covering selection, comparability, and outcome assessment. For studies involving technological development, psychometric validation, or qualitative methods, checklists from the Joanna Briggs Institute (JBI) were applied. Psychometric studies were further evaluated for content validity, internal consistency (e.g., Cronbach’s alpha ≥ 0.80), and statistical robustness.

Reporting guidelines—including the Consolidated Standards of Reporting Trials (CONSORT), Strengthening the Reporting of Observational Studies in Epidemiology (STROBE), and Quality Assessment of Diagnostic Accuracy Studies (QUADAS)—were also used to support methodological appraisal.

All assessments were independently conducted by two reviewers (A.F.d.O. and L.W.P.d.S.) following prior calibration on a pilot set of studies. Discrepancies were resolved by consensus. Risk of bias was classified as high when two or more domains showed “critical risk,” moderate when one critical or multiple concerns were identified, and low otherwise.

### 2.6. Data Synthesis

Given the heterogeneity in study design, population characteristics, technologies evaluated, and outcomes measured, a meta-analysis was not feasible. Instead, a narrative and systematic synthesis was performed in accordance with the Synthesis Without Meta-analysis (SWiM) framework.

To enable structured comparison, studies were grouped by primary type of digital technology: (i) mobile health applications and platforms, (ii) artificial intelligence (AI)-based algorithms and computer vision systems, (iii) wearable sensors and embedded medical devices, and (iv) integrated platforms or clinical decision-support systems. Within each category, outcomes were classified into three domains: clinical outcomes (e.g., ulcer healing, recurrence, glycemic control [HbA1c]); operational outcomes (e.g., treatment adherence, frequency of use, user engagement); and psychometric outcomes (e.g., usability, self-efficacy, perceived usefulness, satisfaction).

Two reviewers independently analyzed and interpreted the findings, with discrepancies resolved through discussion. Summary tables were developed to facilitate cross-category comparisons. Although statistical pooling of effect sizes was not possible, consistent patterns were identified and critically examined, including limitations in external validity and implications for public health policy and digital equity.

## 3. Results

### 3.1. Search Results

The systematic search of PubMed, Scopus, and Web of Science initially identified 95 records. After duplicate removal, 49 unique studies remained. Title and abstract screening excluded 46 records that did not meet the predefined eligibility criteria. The full texts of the remaining 45 studies were then assessed for eligibility, of which 27 were excluded due to lack of clinical or psychometric outcomes, ineligible populations, use of non-digital technologies, or incompatible study designs.

**Figure 1:**
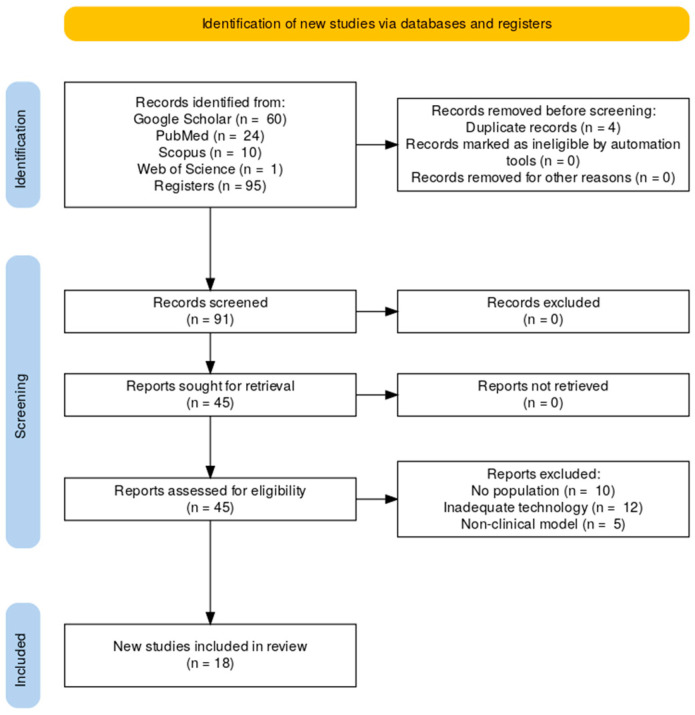
Study selection flowchart through literature search.

In total, 18 studies met all inclusion criteria and were retained for this systematic review [2,12,13,14,15,16,17,18,19,20,21,22,23,24,25,26,27,28]. The selection process, conducted in accordance with the Preferred Reporting Items for Systematic Reviews and Meta-Analyses (PRISMA 2020) guidelines, is illustrated in Figure 1.

### 3.2. Characteristics of the Included Studies

The included populations varied considerably in sample size, age distribution, and clinical complexity. Samples ranged from healthy young adults, primarily recruited for device validation, to individuals with diabetes mellitus presenting with recurrent plantar ulcers, peripheral neuropathy, prior lower-limb amputations, and multiple cardiovascular or metabolic comorbidities. Most studies included middle-aged or elderly participants, reflecting the demographic profile most affected by DFU. Several also reported concomitant medication use, particularly antidiabetic agents (insulin, oral hypoglycemic drugs), cardiovascular therapies (antihypertensives, statins, antiplatelet agents), and treatments for neuropathic pain, all of which may influence ulcer healing, recurrence risk, and adherence to digital interventions.

Sample sizes ranged from single-patient case reports to large image datasets used for computational model training and validation. The digital technologies evaluated were categorized into four groups: (i) mobile health applications and platforms (e.g., INTELLIN^®^, MyFootCare, Minuteful, CARPeDia, Swift Medical Connect); (ii) artificial intelligence (AI) algorithms and computer vision systems (e.g., Faster Region-Based Convolutional Neural Network [R-CNN], Support Vector Machine [SVM], DenseNet, Mask R-CNN, Region-Based Fully Convolutional Network); (iii) wearable sensors and embedded devices, including smart insoles (Orpyx^®^), sensorized boots with inertial measurement units, and plantar thermometry systems (TempStat™); and (iv) clinical decision-support systems, often integrated with electronic medical records and used in outpatient care.

Each category presented distinct strengths and limitations. Mobile applications were widely accessible and user-friendly, improving self-management and adherence, but their effectiveness depended on sustained engagement and digital literacy. AI-based systems achieved the highest technical performance, often surpassing 90% accuracy for ulcer detection and classification, though their generalizability was constrained by small datasets and lack of external validation. Wearable sensors enabled continuous monitoring of plantar pressure and temperature, supporting early detection of recurrence risk, but faced challenges of cost, usability, and long-term adherence. Clinical decision-support systems enhanced professional decision-making when integrated with electronic records, although their direct impact on outcomes remained less validated and depended on interoperability and infrastructure. No single technology emerged as universally superior; instead, complementary or hybrid approaches may offer the most comprehensive solutions for DFU care.

Outcomes were classified into four domains: (i) clinical (ulcer recurrence, progression, new ulcer incidence); (ii) technical (accuracy, sensitivity, specificity, intersection over union [IoU], mean average precision [mAP]); (iii) operational (treatment adherence, user engagement, frequency of image transmission, interaction time); and (iv) psychometric (usability, satisfaction, perceived value).

Of the 18 studies included, 7 employed validated psychometric instruments, such as the System Usability Scale (SUS), EuroQol five-dimension instrument (EQ-5D), Technology Acceptance Model (TAM), self-efficacy scales (Cronbach’s alpha ≥ 0.82), and adapted tools including the National Patient Survey and the OPUS scale [2,12,17,20,23,25,27]. The remaining 11 relied on non-standardized qualitative analyses, ad hoc questionnaires, or lacked structured psychometric assessment, limiting comparability and reproducibility [13,14,15,16,18,19,21,22,24,26,28].

AI-based approaches demonstrated high technical performance, with accuracy above 90% for ulcer detection and classification, comparable to experienced clinical evaluators [16,29]. Nonetheless, most studies used small datasets under controlled conditions, limiting external validity. Performance may vary in real-world clinical environments, where image quality, population heterogeneity, and workflow integration are critical. Large-scale, multicenter validation is therefore required before clinical adoption.

Mobile health applications targeting education and self-care were associated with improved self-efficacy, adherence, and glycemic control, including significant reductions in glycated hemoglobin (HbA1c) [23]. Plantar sensor technologies proved effective for early detection of pressure and temperature changes, enabling timely interventions such as offloading and footwear adjustments [14,25]. However, long-term adherence was inconsistent, influenced by comfort, device durability, and daily usability. Most studies also involved small samples and short follow-up, limiting evidence strength. Still, plantar sensors hold promise as cost-effective adjuncts to clinical monitoring in high-risk populations.

Key methodological limitations included socioeconomic barriers (restricted access to mobile devices, poor connectivity), low digital literacy, technical failures in system interoperability, and small sample sizes that undermined statistical power and generalizability [8,23,30,31,32,33,34].

Table 1 presents a summary of the key methodological characteristics and empirical findings of the studies included in this systematic review.

### 3.3. Quality Assessment

Among the randomized clinical trials (*n* = 3), Chen et al. [23] and Hellstrand Tang et al. [27] reported adequate randomization and partial blinding, minimizing potential performance bias. Only Chen et al. [23] explicitly described the use of intention-to-treat analysis. The protocol by Lazo-Porras et al. [14] was considered eligible; however, as no outcome data were available, a full methodological appraisal was not possible.

For studies focused on the development and validation of digital technologies (*n* = 9), quality appraisal considered detailed reporting of system architectures, use of cross-validation techniques (e.g., k-fold, external validation), and robust performance metrics such as accuracy, sensitivity, specificity, intersection over union (IoU), and mean average precision (mAP) [2,13,15,16,17,18,22,24,25]. Six studies applied either cross-validation or external testing, while three provided comprehensive descriptions of modeling strategies. Notably, Goyal et al. [16] and Ferreira et al. [2] explicitly acknowledged algorithmic limitations, enhancing transparency and reproducibility in computational analyses.

Studies evaluating wearable sensors or embedded medical devices (*n* = 4) [18,19,25,28] emphasized in-field clinical validation, use of objective indicators of user engagement (e.g., duration of use, number of alerts generated), and integration with mobile platforms or electronic health records. Park et al. [25] and Matijevich et al. [28] reported the most rigorous protocols in this category, although heterogeneity in engagement metrics limited direct comparability.

In usability and perception studies (*n* = 6) [2,12,17,23,25,27], validated psychometric instruments were frequently applied, including the System Usability Scale (SUS), Technology Acceptance Model (TAM), EuroQol five-dimension instrument (EQ-5D), and adapted tools such as the National Patient Survey and the OPUS scale. Mean scores consistently exceeded 80, indicating high acceptability. In contrast, two studies relied solely on non-standardized qualitative methods or ad hoc questionnaires and were thus classified as presenting a moderate risk of descriptive bias [19,26].

One study (*n* = 1), Haycocks et al. [21], employed a mixed-methods design that combined quantitative outcomes (e.g., SINBAD score, cost–utility analysis using Markov modeling) with qualitative usability evaluation. However, the absence of validated psychometric instruments and a formal methodological framework for qualitative analysis led to classification as moderate risk.

Overall, studies were stratified into three levels of bias risk: (i) low risk, assigned to 13 investigations with robust design, validated outcome measures, and well-defined populations (e.g., Chen et al. [23], Ferreira et al. [2], Zoppo et al. [18]); (ii) moderate risk, assigned to four studies with limitations such as small sample sizes, absence of control groups, or reliance on unvalidated instruments (e.g., Ploderer et al. [19], Kong et al. [24], Keegan et al. [26], Haycocks et al. [21]); and (iii) high or undefined risk, attributed to the protocol by Lazo-Porras et al. [14] and to studies that did not adequately report psychometric assessment strategies.

Table 2 provides a structured synthesis of methodological validation, use of psychometric instruments, and risk-of-bias assessment across all included studies, complementing the narrative description and facilitating rapid cross-study comparison.

## 4. Discussion

To our knowledge, this is the first systematic review to comprehensively synthesize evidence on digital health technologies applied to the prevention, detection, and management of diabetic foot ulcers (DFU). The findings indicate that mobile applications, wearable sensors, artificial intelligence (AI)-based algorithms, and integrated platforms hold considerable potential to enhance diagnostic accuracy, promote patient engagement, and enable remote lesion monitoring across diverse care settings.

AI-based interventions emerged as the most technically advanced. Studies employing convolutional neural networks (CNNs)—including Faster R-CNN, Mask R-CNN—and DenseNet architectures reported sensitivity and specificity above 90%, approaching or surpassing the diagnostic performance of clinical specialists. Despite these promising results, most studies were limited by small datasets, absence of external validation, and scarce evidence of real-world implementation. Variability in image quality, device resolution, and workflow integration further constrained generalizability. Standardization of datasets and adherence to frameworks such as CONSORT-AI and STARD-AI are essential to strengthen reproducibility and reliability.

Mobile applications demonstrated improvements in self-care, treatment adherence, and glycemic control, particularly when grounded in behavioral models such as Bandura’s Theory of Self-Efficacy. For example, Chen et al. [23] reported significant increases in self-efficacy scores and reductions in glycated hemoglobin among users of an educational platform. These tools are low-cost and accessible, making them attractive in public health contexts. However, their effectiveness depends heavily on sustained engagement and digital literacy. Socioeconomic inequalities, lack of device compatibility, and poor internet connectivity remain critical barriers, especially in low-resource and older populations. Gaps in digital literacy, rural connectivity, and affordability further reinforce inequities in access.

Sensor-based technologies, including smart insoles and thermal or pressure monitoring systems, provided continuous and objective detection of plantar load and temperature variations—risk factors strongly associated with ulcer recurrence. Studies such as Matijevich et al. [28] reported recurrence prevention benefits during longitudinal follow-up. Nonetheless, limited sample sizes, lack of control groups, non-standardized adherence measures, and issues of cost, comfort, and durability reduced scalability. Long-term adherence was frequently undermined by integration challenges with daily routines.

Emerging imaging modalities, such as infrared thermography and automated wound measurement, remain exploratory but show potential for broader integration. For instance, Wijesinghe et al. [16] developed an AI-powered telehealth prototype that combined retinal and DFU imaging, achieving >97% classification accuracy. Despite strong technical performance, these approaches remain underrepresented in the literature, warranting further validation in diverse clinical contexts.

Assessments of usability and user perception revealed substantial methodological heterogeneity. While some studies employed validated instruments such as the System Usability Scale (SUS), others relied on non-standardized questionnaires or qualitative assessments, limiting comparability. Haycocks et al. [21], for example, relied exclusively on non-validated self-reported data, while Ploderer et al. [26] applied Likert scales without psychometric validation. These weaknesses underscore the importance of employing culturally adapted and validated tools, particularly for low-literacy and digitally vulnerable populations.

Geographical disparities were also evident. Most studies originated from high-income countries (e.g., United States, United Kingdom, Canada), where research focused primarily on technical validation and usability under controlled conditions. In contrast, studies from low- and middle-income countries (LMICs), such as Brazil and Peru, emphasized barriers related to infrastructure, health equity, and system integration, particularly within universal health systems such as the Brazilian Unified Health System (SUS). These systems face persistent challenges, including the absence of standardized national digital health protocols, fragmented electronic infrastructures, and insufficient policy frameworks for innovation.

Another critical gap is the paucity of robust economic evaluations. Only a minority of studies assessed cost-effectiveness, cost–utility, or organizational impact in real-world settings. Such analyses are crucial for decision-makers in resource-constrained contexts, where evidence of economic value is essential to justify technology adoption and guide rational allocation of scarce resources. Without this, innovations risk remaining confined to pilot projects.

Comparative synthesis across technology categories suggests complementary strengths and limitations. Mobile applications are accessible but dependent on literacy and engagement. AI systems achieved the highest diagnostic accuracy (>90%) but require larger, more diverse datasets and multicenter validation. Wearable sensors provide continuous monitoring but face adherence, usability, and cost barriers. Imaging-based modalities remain exploratory but present opportunities for integration into hybrid digital ecosystems. No single technology emerged as universally superior; instead, hybrid or integrated strategies appear most promising for comprehensive, equitable, and sustainable DFU management.

Despite the enthusiasm surrounding digital health, this review identified significant methodological limitations. Few studies employed randomized controlled designs or intention-to-treat analyses, and statistical modeling was often limited. The wide variation in outcomes—spanning computational metrics (e.g., accuracy, sensitivity), clinical endpoints (e.g., recurrence, healing time), and psychometric measures (e.g., usability, self-efficacy)—precluded meta-analysis and hampered comparability. Furthermore, while age and medication use were frequently reported, few studies explored their influence on technology adoption, adherence, or clinical outcomes, representing critical gaps for future research.

Methodological quality appraisal indicated that most studies presented low or moderate risk of bias. Investigations with robust designs, validated outcomes, and well-defined populations (e.g., Chen et al. [23], Ferreira et al. [2], Zoppo et al. [18]) were classified as low risk. Conversely, studies with small samples, lack of control groups, or reliance on unvalidated instruments (e.g., Kong et al. [19], Ploderer et al. [26], Haycocks et al. [21]) were deemed moderate risk. The protocol by Lazo-Porras et al. [14] was considered high or undefined risk due to the absence of outcome data.

Among the strengths of this review are the comprehensiveness of the search strategy, critical appraisal stratified by technology type, outcome domain, and study design, and the use of validated tools for bias assessment across diverse methodological frameworks. Limitations include the exclusion of studies in non-Latin languages and heterogeneity in reporting standards, which limited comparability. Future reviews should adopt mixed-methods approaches and advanced synthesis frameworks such as SWiM (Synthesis Without Meta-analysis), supplemented by sensitivity analyses.

In summary, digital health technologies represent a transformative frontier in DFU management. Yet their integration into clinical practice requires more than technical validation: it demands investment in digital infrastructure, development of standardized protocols, establishment of regulatory frameworks, and robust economic and implementation studies. Only through such measures can these innovations be scaled equitably and sustainably within real-world health systems.

## 5. Conclusions

Digital health technologies for diabetic foot ulcer (DFU) care have shown promising results in both prevention and remote monitoring. Artificial intelligence (AI)–based tools achieved high diagnostic accuracy in detecting and segmenting foot ulcers, while mobile health applications—particularly those grounded in behavioral models—improved self-efficacy, self-care behaviors, and glycemic control. Wearable sensors integrated into digital platforms demonstrated potential for reducing ulcer recurrence among high-risk patients.

Nevertheless, significant gaps remain. Most studies were limited by small sample sizes, lack of control groups, absence of external validation for computational models, and reliance on non-standardized psychometric instruments. These methodological weaknesses reduce robustness and reproducibility, restricting safe translation into real-world clinical practice.

Future research should prioritize randomized controlled trials, long-term follow-up, and comprehensive economic evaluations. Importantly, digital interventions must be adapted to the structural and operational realities of public health systems, particularly in low- and middle-income countries (LMICs), where inequities and resource constraints are most pronounced. In this context, health authorities operating under universal health coverage models—such as the Brazilian Unified Health System (SUS)—should advance a strategic agenda for digital health integration. This includes investments in interoperable and scalable platforms integrated with health information systems, capacity-building initiatives to strengthen digital literacy and care coordination in primary care, and the development of regulatory frameworks and public policies that promote equitable access to digital tools for vulnerable populations.

Such measures are essential not only to foster technological innovation but also to ensure that digital health functions as a mechanism for inclusion, access, and quality care—rather than as an additional driver of exclusion—within health systems committed to universality, equity, and comprehensiveness.

## Figures and Tables

**Table 1:** Characteristics of the included studies.

Authors/Year	Study Location	Study Design	Population	Technology Used	Outcomes Assessed	Psychometric Instruments	Main Findings
Hazenberg et al./2012 [12]	Netherlands and Germany	Prospective feasibility study with four-month home follow-up	22 patients with DM1 or DM2, peripheral neuropathy and plantar deformities; mean age 60 years	The Portable Foot Imaging Device (PFID) provides high-resolution plantar imaging with automatic data transmission via modem.	Technical feasibility, clinical utility of images, quality of life and usability	EQ-5D, VAS (0–10) for usability	High lesion detection rate; usability VAS 7–9; slight improvement in EQ-5D; use <6 min
Wang et al./2015 [13]	USA	Methodological study with experimental evaluation of system	30 simulated and 34 real wounds in patients with DM2	Android app with optical box, automatic segmentation via Mean-Shift and K-means (GPU acceleration)	Segmentation accuracy, RYB classification, processing time	Validation by three experts, MCC index	Satisfactory segmentation (MCC = 0.736); average time of 15s per image; viable for near real-time use
Lazo-Porras et al./2016 [14]	Lima, Peru	Randomized, controlled clinical trial, with blinding of the evaluator (protocol)	Adults (18–80 years) with T2DM, risk 2 or 3 (IWGDF), pedal pulse present, with cell phone and consent	TempStat™ (plantar thermometry) + SMS/audio sending with self-care guidance	Primary: 12-month ulcer incidence; Secondary: TempStat™ adherence, engagement, and thermal response	Not applicable	Not applicable (study protocol)
Wang et al./2017 [15]	USA	Technological development and initial validation (computer system)	Real clinical images of ulcers captured via smartphone (number of patients not specified)	ML system: superpixels (SLIC), color descriptors, texture, DSIFT-BoW, and two-stage SVM	Accuracy in ulcer segmentation, feasibility on smartphones for remote monitoring	Not applicable	High accuracy in automated detection; feasibility on mobile devices; reduction in false positives/negatives
Goyal et al./2019 [16]	United Kingdom	Methodological study of development and validation of predictive models with deep learning	1775 images of feet with ulcers and 105 healthy ones	Faster R-CNN (InceptionV2), SSD (InceptionV2/MobileNet), R-FCN (ResNet101) with transfer learning	Accuracy, speed and IoU for real-time DFU detection	Not applicable	Faster R-CNN InceptionV2: mAP 91.8%, IoU 95.5%, 48 ms/img; SSD-MobileNet: 30 ms, mAP 83.6%; 80% accuracy via Jetson TX2/Android
Wijesinghe et al./2019 [17]	Sri Lanka	Development and evaluation of prototype with technical validation and usability	5 experts and 10 participants	Prototype consisting of a smartphone-based application (IDA app) integrated with a cloud telehealth platform, combined with deep learning algorithms (DenseNet-201, ResNet-18, VGG-16) and Mask R-CNN for image analysis; hardware included smartphone camera and data transmission modules	Accuracy in DR and DFU classification, segmentation, image retrieval, usability	System Usability Scale (SUS)	Accuracy >98% (DR), >97% (DFU); mAP >87% (segmentation), >99% (retrieval DR); SUS 88.5; better than 5 clinical
Zoppo et al./2020 [18]	Italy	Prospective, observational, comparative, non-randomized and monocentric clinical study	150 patients with chronic wounds (vascular, DFU and pressure)	Wound Viewer: AI with IR sensors, CMOS camera, LEDs, DT-CNN algorithm; AWS integration with GDPR/HIPAA	Area, depth, volume, WBP, tissue segmentation, diagnostic accuracy and comparison with other methods	Falanga WBP; confusion matrix; Kruskal–Wallis and Kolmogorov–Smirnov tests	97% bedside accuracy; measurements equivalent to conventional measurements (*p* = 0.9); error <14%; necrosis detected ≥7.3%; safe and non-invasive remote monitoring
Kong et al./2021 [19]	Canada	Clinical case study (case report)	Man, 57 years old, DM1, chronic ulcer, osteomyelitis, multiple comorbidities (CAD, CKD, PAD, previous amputation)	Swift Medical App—Patient Connect (Computer Vision, Calibrated Images, Encrypted Data, HIPAA/FDA Compliant)	Primary: wound evolution; Secondary: adherence, reduced consultations, cost/time, self-care, infection management	Not applicable	Images sent increased (2→39); effective control of 3 infections; reduction in time (~3 h) and cost (~US$50/visit); patient reported platform as educational and empowering
Bahaadinbeigy et al./2022 [20]	Iran	Methodological study in four phases (development and evaluation)	15 experts (Delphi) and 4 healthcare professionals (usability)	Telemedicine system in ASP with SQL database and SSL security protocol	Information needs, system usability, user satisfaction	Validated questionnaire (α = 0.952) + satisfaction questionnaire by experts	System with 75 essential items (registration, prescription, communication); 26 usability problems identified
Haycocks et al./2022 [21]	United Kingdom	Prospective feasibility study, mixed approach	15 patients with DM and healed diabetic foot ulcer	INTELLIN^®^ (mHealth) app with monitoring, engagement and Markov model for cost–utility	Ulcer recurrence, SINBAD score, self-reference, usability, cost-effectiveness	Qualitative collection without validated instruments	53% with relapse (mean 273 days), mean SINBAD 2.1, no self-referral, high usability, ICER £20,000/QALY with ≥5% reduction in relapse, socioeconomic barriers limited adherence
Cassidy et al./2023 [22]	United Kingdom and New Zealand	Multicenter, prospective, observational, clinical proof-of-concept study	81 patients with diabetes; 203 images (162 with ulcer, 41 without)	Low-cost smartphone-embedded AI for automated ulcer detection	Sensitivity, specificity, reliability (Kα)	Krippendorff’s Kappa (Kα > 0.80) for AI agreement vs. human raters	Sensitivity 91.6%, specificity 92.4%, high Kα; performance comparable to clinical; feasibility of automated remote monitoring
Chen et al./2023 [23]	Taiwan	single-blind clinical trial	100 elderly people with DM2 (average age 67.6 years); 50 control and 50 intervention	Digital self-care program based on Self-Efficacy Theory with videos, games, LINE messages and calls	Self-efficacy, foot self-care, HbA1c	Self-Efficacy Scale (α = 0.82) and Self-Care Scale (α = 0.92), Chinese version	Significant improvement in intervention: self-efficacy (24.96→76.56), self-care (8.08→32.36), HbA1c reduction by 0.41% (*p* < 0.001); control with less improvement
Ferreira et al./2023 [2]	Brazil	Methodological study with development and validation of neural network and application	250 for training/validation and 141 for testing; all with DM in the APS of Minas Gerais	MLP neural network integrated into the CARPeDia app (JavaScript); 10 × 10 × 2 architecture	Accuracy, sensitivity, specificity, PPV, NPV; usability (SUS)	Cross-validation (10-fold), Friedman test, Dunn–Bonferroni; SUS (93.3/100)	Accuracy 85%, sensitivity 84%, specificity 89%; high usability; parsimonious and applicable model with customized report generation
Keegan et al./2023 [24]	Baltimore, USA	Prospective, quantitative pilot study with technological intervention (8 weeks)	25 patients with DFU and history of revascularization/podiatry; mean age 65.5 years; 60% men; 52% black	Healthy.io Minuteful app, a smartphone-based wound imaging system using calibration markers for standardized images, automatic cloud upload, and AI-driven analysis providing wound size/healing progression reports; enabled remote monitoring and patient self-scanning	Engagement, satisfaction, therapeutic approach, wound reduction, healing, failures and technical support	Non-validated instrument (Likert + open questions developed by the team)	84% adhered to ≥1 scan, 20% completed all; 36% had adjusted conduct; mean wound reduction 41.6% (*p* = 0.005); 12% healed; 94.1% approved; technical and socioeconomic barriers
Park et al./2023 [25]	Texas, USA	Experimental study of technological validation (pilot)	14 healthy adults (mean age 31.6 ± 8.7 years; 64% women)	Orthopedic boot with IMU sensors, smartwatch, cloud-based clinical dashboard, wearable sensors for balance/gait	Adherence to use, postural stability (COM sway), step count, usability and acceptance	Adapted TAM questionnaire (5-point Likert, Q1–Q9)	Grip accuracy: 89.3%; improved stability (*p* < 0.05); step counting errors: 4.4% (slow), 36.2% (normal), 16% (fast); high acceptance, except aesthetics
Ploderer et al./2023 [26]	Australia	Prospective mixed methods study (predominantly qualitative), 3 months	12 patients with plantar ulcers (DM1/DM2), caregivers, access to Android smartphone	MyFootCare App (Android) with OpenCV and watershed algorithm for photo segmentation	Perception of value, engagement, barriers/facilitators to use and applicability	No validated psychometric scales applied; evaluation based on qualitative interviews and ad hoc Likert ratings (1–10) at weeks 0, 3, and 12	App perceived as useful; usage varied; facilitators: familiarity and support; barriers: usability, low digital literacy, limited image accuracy
Hellstrand et al./2024 [27]	Sweden	Randomized, patient-blinded, two-arm parallel clinical trial	100 patients with DM (47 intervention, 53 control; mean 66 ± 13 years), 2 evaluators (ort/prot)	CDSS for foot examination compared to traditional clinical examination	Patient satisfaction, professional experience, clinical interaction	National Patient Survey (modified) and OPUS	High satisfaction in both; OPUS without difference (*p* = 0.78); good usability; preserved professional-patient interaction
Matijevich et al./2024 [28]	USA	Prospective cohort study with illustrative case series	3 patients with T2DM, peripheral neuropathy, history of ulcers; ages 49–75; 2 with amputations	Orpyx^®^: sensory insoles with pressure, temperature and IMU sensors, with biofeedback via app	Plantar pressure, thermal variation, pre-ulcerative lesions, engagement and need for intervention	Adherence estimated by usage time, tracked steps, inactivity alerts, and interactions via RPM	No new ulcers in 8 months; pressure guided adjustments; temperature alone was insensitive; combined approach reinforced prevention and avoided recurrence

**Table 2:** Sintese Quality assessment.

Study	Type of Study	Methodological Validation	Use of Psychometric Instruments	Bias Assessment
Hazenberg et al., 2012 [12]	Feasibility study with sensors	EQ-5D, time of use, practical evaluation	EQ-5D; VAS	Low
Wang et al., 2015 [13]	Algorithm with optical box	MCC; expert testing	Not applicable	Low
Lazo-Porras et al., 2016 [14]	Clinical trial protocol	Protocol without final data	Not applicable	High/Undefined
Wang et al., 2017 [15]	Automated segmentation system	High accuracy; technical validation	Not applicable	Low
Goyal et al., 2019 [16]	Deep learning with external validation	Detailed architecture and mAP/IoU	Not applicable	Low
Wijesinghe et al., 2019 [17]	AI-powered telehealth	mAP, classification superior to clinical	SUS	Low
Zoppo et al., 2020 [18]	Comparative clinical study	Comparison with gold standards	WBP, robust statistical analysis	Low
Kong et al., 2021 [19]	Clinical case report	Narrative drawing	Not applicable	Moderate
Bahaadinbeigy et al., 2022 [20]	Telemedicine system (Delphi)	4-phase assessment; validated questionnaire	Cronbach’s alpha = 0.952	Low
Haycocks et al., 2022 [21]	Feasibility study with mixed approach	SINBAD score, absence of self-reference, economic analysis with Markov model	Non-standardized qualitative analysis	Moderate
Cassidy et al., 2023 [22]	Computer Vision and AI	Kappa, sensitivity, specificity	Kα (Krippendorff)	Low
Chen et al., 2023 [23]	Randomized clinical trial	Randomization and partial blinding; ITT mentioned	SUS and validated scales	Low
Ferreira et al., 2023 [2]	Technological validation with RNAs	Cross-validation and statistical testing	SUS	Low
Keegan et al., 2023 [24]	Pilot study of digital intervention	Clinical and operational results	Questionnaire not validated	Moderate
Park et al., 2023 [25]	Technological validation with sensors	Accuracy, engagement, technical validation	Adapted TAM	Low
Ploderer et al., 2023 [26]	Qualitative study with app	Thematic analysis without structured method	Likert 1–10; no validated scale	Moderate
Hellstrand et al., 2024 [27]	Randomized clinical trial	Randomization and partial blinding	OPUS; Modified national scale	Low
Matijevich et al., 2024 [28]	Prospective cohort with sensors	Continuous collection, engagement, adherence	Adherence via objective data	Low

## Supplementary Materials

The following supporting information can be downloaded at: https://www.mdpi.com/article/10.3390/ijerph22091430/s1, File S1: Search criteria for articles in the databases.
