# Supplementary material for: Digital Health Technologies for Diabetic Foot Ulcers: A Systematic Review of Clinical Evidence, Access Inequities, and Public Health Integration
Source: Int J Environ Res Public Health. 2025 Sep 13;22(9):1430. doi: 10.3390/ijerph22091430 (PMC12469766; doi:10.3390/ijerph22091430)
Supplement: Supplementary file 1 [file ijerph-22-01430-s001.zip › ijerph-3802053-supplementary.pdf]

International Journal of
Environmental Research
and Public Health

MDPI

Suplementary Material 1

Pubmed:

#1 "Diabetic Foot"[Mesh] OR "Foot Ulcer"[Mesh] OR "Foot Injuries"[Mesh] OR "diabetic foot" OR "foot ulcer" OR "foot ulcers" OR "foot lesion*" OR "foot complication*"

#2 "Diabetes Mellitus"[Mesh] OR "type 1 diabetes" OR "type 2 diabetes" OR "diabetes mellitus"

#3 "Telemedicine"[Mesh] OR "Mobile Applications"[Mesh] OR "Remote Consultation"[Mesh] OR "eHealth" OR "mHealth" OR "digital health" OR "digital technology" OR "mobile app*" OR "smartphone application*" OR "electronic monitoring" OR "digital tool*" OR "mobile health" OR "health app*"

#4 "Validation Study"[Publication Type] OR "Psychometrics"[Mesh] OR "validation" OR "reliability" OR "validity" OR "questionnaire validation" OR "instrument validation" OR "construct validity" OR "content validity" OR "reproducibility of results"

#5 "Prevention"[Mesh] OR "Early Diagnosis"[Mesh] OR "early detection" OR "screening" OR "preventive care" OR "monitoring"

#6 #1 AND #2 AND #3 AND #4 AND #5

#7 "review"[Publication Type] OR "editorial"[Publication Type] OR "letter"[Publication Type] OR "comment"[Publication Type]

#8 #6 NOT #7

SCOPUS

TITLE-ABS-KEY("diabetic foot" OR "foot ulcer" OR "foot ulcers" OR "foot lesion*" OR "foot complication*") AND TITLE-ABS-KEY("diabetes mellitus" OR "type 1 diabetes" OR "type 2 diabetes") AND TITLE-ABS-KEY("digital health" OR "digital technolog*" OR "telemedicine" OR "mobile application*" OR "mobile app*" OR "eHealth" OR "mHealth" OR "remote monitoring" OR "electronic monitoring" OR "smartphone application*" OR "health app*") AND TITLE-ABS-KEY(validation OR "validation study" OR "instrument validation" OR "questionnaire validation" OR "psychometric*" OR "construct validity" OR "content validity" OR reliability OR reproducibility) AND TITLE-ABS-KEY(prevention OR "preventive care" OR "early detection" OR "early diagnosis" OR monitoring OR screening) AND NOT DOCTYPE("re" OR "ed" OR "le" OR "sh" OR "no" OR "er")

WEB OF SCIENCE

#1 TS=("diabetic foot" OR "foot ulcer" OR "foot ulcers" OR "foot lesion*" OR "foot complication*")

#2 TS=("diabetes mellitus" OR "type 1 diabetes" OR "type 2 diabetes")

#3 TS=("digital health" OR "digital technolog*" OR telemedicine OR "mobile application*" OR "mobile app*" OR eHealth OR mHealth OR "remote monitoring" OR "electronic monitoring" OR "smartphone application*" OR "health app*")

#4 TS=(validation OR "validation study" OR "instrument validation" OR "questionnaire validation" OR psychometric* OR "construct validity" OR "content validity" OR reliability OR reproducibility)

#5 TS=(prevention OR "preventive care" OR "early detection" OR "early diagnosis" OR monitoring OR screening)

#6 #1 AND #2 AND #3 AND #4 AND #5

GOOGLE SCHOOLAR

("diabetic foot" OR "foot ulcer" OR "foot ulcers" OR "foot lesion" OR "foot complications") AND ("diabetes mellitus" OR "type 1 diabetes" OR "type 2 diabetes") AND ("digital health" OR "telemedicine" OR "mobile application" OR "mobile app" OR "eHealth" OR "mHealth" OR "remote monitoring" OR "electronic monitoring" OR "smartphone application" OR "health app") AND ("validation" OR "validation study" OR "instrument validation" OR "questionnaire validation" OR "psychometric" OR "construct validity" OR "content validity" OR "reliability" OR "reproducibility") AND

("prevention" OR "preventive care" OR "early detection" OR "early diagnosis" OR "monitoring" OR "screening")
